# B and NK Cells Closely Correlate with the Condition of Patients with Acute Pancreatitis

**DOI:** 10.1155/2019/7568410

**Published:** 2019-02-06

**Authors:** Xin Wei, Weifeng Yao, Huiping Li, Jingjing Qian, Yaosheng Xie, Zhuo Zhang, Hong Lu, Liang Shi, Xiangyang Lin

**Affiliations:** ^1^Department of Clinical Laboratory, The First Affiliated Hospital of Wenzhou Medical University, Wenzhou, China; ^2^Department of Key Laboratory of Diagnosis and Treatment of Severe Hepato-Pancreatic Diseases of Zhejiang Province, The First Affiliated Hospital of Wenzhou Medical University, Wenzhou, China

## Abstract

**Purpose:**

Pancreatitis can lead to systemic inflammatory response, but the relationship between lymphocyte changes and patients with pancreatitis remains unclear. In this study, we evaluated the feedback function of changes in peripheral lymphocyte subsets on the condition of patients with pancreatitis.

**Materials and Methods:**

131 acute pancreatitis (AP) patients and 11 chronic pancreatitis (CP) patients constituted the patients' group; 20 healthy individuals were enrolled as healthy controls (HC). Serum concentration of C-reactive protein (CRP), amylase, and lipase and the frequency and absolute number of many types of peripheral lymphocytes (including T, B, NK, CD16+/CD56+ T, CD4+ T, CD8+ T, CD4+CD8+ T, and CD4−CD8− T cells) were detected on admission and the seventh day of standard treatment. Besides, the length of hospital stay was recorded.

**Results:**

The absolute number of all lymphocytes we studied decreased in patients with CP and in patients with almost all types of AP. The frequency change of lymphocytes varies among the different types of AP. During disease onset, B cell frequency correlated positively with CRP concentration and NK cell frequency correlated positively with amylase and lipase concentration. B cell frequency and CD4+ T cell absolute number were recovering towards normal after short-term treatment. The frequency of B cells and NK cells correlated positively with the length of hospital stay.

**Conclusions:**

B cells and NK cells closely correlate with patients' condition and may help to diagnose AP more accurately and reflect treatment effect of AP in time, affecting the recovery speed of patients with M-AP, which may help physicians to better understand the pathophysiology of pancreatitis.

## 1. Introduction

Acute (AP) and chronic (CP) pancreatitis are pancreas inflammatory response that can be induced by a variety of factors including cholelithiasis, biliary blockage, alcohol, hyperlipidemia, autoimmunity, and other nonspecific factors [1, 2]. According to the severity, AP can be classified as mild AP (M-AP) and severe AP (S-AP) [3]. If AP is not accurately diagnosed in time, it may delay unhealed, leading to systemic inflammatory response and multiorgan failure, threating life [1, 4, 5].

Lymphocytes act as important immunoregulatory cells and can secrete various cytokines to directly or indirectly regulate immune response. It has been reported that activated T cells and B cells play an important regulatory role in various inflammatory responses including pancreatitis [6]. Peripheral lymphocytes have undergone momentous changes under the condition of pancreatitis. Pietruczuk et al. [7] revealed that there was a group of significantly activated lymphocytes in AP patients with enhanced ability to secrete Th2-type cytokines. In addition, increased monocytes and reduced apoptosis-induced NK cells and CD4+ T cells were found in early AP [8].

The diagnosis of AP and CP is still more certain with the aid of computed tomography, ultrasonography, and some biochemical indicators including amylase and lipase [2]. However, the value of changes in peripheral lymphocyte subsets for the diagnosis and prognosis of AP and CP remains unclear. In this study, we did a dynamic monitoring on peripheral lymphocyte subsets before and after a standard treatment; also, the indicators (CRP, amylase, and lipase) which highly correlate with pancreatitis were monitored throughout the study. In addition, we performed a correlation analysis to find out the value of changes in lymphocyte subsets on auxiliary diagnosis and disease control of pancreatitis and its feedback function on therapeutic efficacy. Furthermore, we analyzed the relationship between the change of peripheral lymphocyte subsets at admission and the recovery speed of patients with pancreatitis.

## 2. Materials and Methods

### 2.1. Study Subjects

131 AP and 11 CP patients were enrolled for this study in the First Affiliated Hospital of Wenzhou Medical University between August 2017 and January 2018. AP was diagnosed according to the following criteria: abdominal pain (acute onset of persistent and severe epigastric pain, often radiating to the back), serum lipase (or amylase) activity at least three times the upper limit of normal (lipase: 5-60 U/L; amylase: 28-100 U/L), or characteristic findings of AP on contrast-enhanced CT or, less often, MRI or transabdominal ultrasonography [9]. The severity of AP was defined according to the Atlanta criteria [10] and serum CRP concentration. The diagnosis of CP is based on a combination of clinical symptoms, including abdominal pain, exocrine insufficiency, fat maldigestion and steatorrhea, carbohydrate and protein maldigestion, and endocrine insufficiency, and confirmed by morphologic, functional, and/or histologic criteria [11]. Twenty age-matched and sex-matched healthy individuals were enrolled as healthy controls (HC, male/female: 8/12, age: 47.60 ± 2.552). Main information about the patients is in Tables 1 and 2. In all patients, the time between abdominal pain onset and admission to the hospital was not longer than 48 h. To investigate the relationship between changes in peripheral lymphocyte subsets and the therapeutic efficacy and disease control of pancreatitis, we randomly selected 79 patients from the 131 AP patients enrolled, including 68 M-AP patients and 11 S-AP patients, detected the changes in lymphocytes after a 7-day standard treatment; main information about the patients is in Table 3. The study protocol was approved by the ethics committee of our unit, and written informed consent was obtained from each subject.

### 2.2. Treatment

All patients were treated according to a standardized interdisciplinary management protocol including a combination of traditional Chinese medicine and Western medicine. The treatment protocol includes nonsurgical treatment, treatment for the cause, and surgical treatment. Nonsurgical treatment includes (1) general treatment, including fasting, gastrointestinal decompression, antispasmodic, analgesic, protease inhibitors, and trypsin inhibitor treatment; (2) fluid resuscitation and intensive care treatment, resuscitation fluid preferred lactated Ringer's solution, for patients requiring rapid resuscitation, appropriate plasma preparations are chosen; (3) maintenance treatment of organ functions, including treatment for respiratory failure, treatment for acute renal failure, and other organ function support; (4) nutritional support, once intestinal function is restored, enteral nutrition should be performed as soon as possible; (5) antibiotic application, for patients with AP, intravenous antibiotics are not recommended to prevent infection and for some susceptible people (such as biliary obstruction, advanced age, and low immunity), enterogenous bacterial translocation may occur and quinolones, cephalosporins, carbapenems, and metronidazole may be selected to prevent infection; and (6) traditional Chinese medicine treatment, Chinese medicine can be used to promote the recovery of gastrointestinal function and the absorption of pancreatic inflammation, including oral administration, external application, or enema. Treatment for the cause includes treatment for (1) biliary acute pancreatitis, all patients with biliary obstruction need to remove the obstruction in time, treatment includes endoscopic or surgical treatment; (2) hyperlipidemia acute pancreatitis, using low molecular weight heparin and insulin or lipid adsorption and plasma exchange to rapidly reduce lipid loading; and (3) other causes, alcoholic pancreatitis patients need to quickly eliminate alcohol, to prevent alcohol intake. AP caused by pancreas anatomy, physiological abnormalities, and drugs is treated accordingly. Surgical treatment is mainly for pancreatic local complications with secondary infection or compression symptoms, such as digestive tract obstruction, biliary obstruction, pancreatic fistula, digestive tract fistula, and pseudoaneurysm rupture. The treatment protocol is in line with the guidelines for the diagnosis and treatment of acute pancreatitis (2014) made by the Chinese Medical Association Surgery Branch Pancreatic Surgery Group. And all patients were discharged when discharge criteria are met according to Chinese discharge standards.

### 2.3. Blood Sampling and Laboratory Procedure

Whole blood specimens were obtained from all subjects under sterile conditions on admission and on the seventh day of standard treatment. Blood samples were drawn from the antecubital vein. 2 mL EDTA-anticoagulated blood sample was used to detect the frequency and absolute number of lymphocyte subsets (total T, CD4+ T, CD8+ T, CD4+CD8+ T (DP T), CD4−CD8− T (DN T), B, NK, CD3+CD16+, and/or CD56+ T (NKT) cells) by flow cytometry. 8 mL blood was collected into procoagulation tubes and centrifuged for serum to test biochemical indicators, including CRP, amylase, and lipase. Cell cluster of differentiation antigens (CD) were assayed by flow cytometry using commercially available procedures (The BD Multitest IMK kit: BD Multitest™ CD3/CD8/CD45/CD4 reagent and BD Multitest™ CD3/CD16+CD56/CD45/CD19 reagent. These antibodies are labeled as follows: PerCP-anti-CD45, FITC-anti-CD3, APC-anti-CD4, PE-anti-CD8, APC-anti-CD19, PE-anti-CD16, and PE-anti-CD56; all fluorescence antibodies were purchased from BD Biosciences (San Jose, CA, USA)). Briefly, 100 *μ*L samples of the whole blood was incubated with 10 *μ*L of the BD Multitest™ CD3/CD8/CD45/CD4 reagent solution or BD Multitest™ CD3/CD16 + CD56/CD45/CD19 reagent solution. After 15 minutes of incubation in the dark at room temperature, erythrocytes were lysed, while leukocytes were fixed and stabilized, and the solution was washed twice with PBS. Then cells were analyzed by BD FACSCanto II (Becton Dickinson, San Jose, CA, USA) and FlowJo software (Tree Star, Ashland, OR, USA). The gate strategy was executed as follows: T cells: CD45+CD3+, B cells: CD45+CD3−CD19+, NK cells: CD45+CD3−CD56+ and/or CD16+, NKT cells: CD45+CD3+CD56+ and/or CD16+, CD4+ T cells: CD45+CD3+CD4+CD8−, CD8+ T cells: CD45+CD3+CD4−CD8+, DP T cells: CD45+CD3+CD4+CD8+, and DN T cells: CD45+CD3+CD4−CD8−. The results of lymphocyte subsets are reported as percentages accounted for total lymphocytes.

### 2.4. Statistical Analysis

Data are expressed as the mean ± SEM for each group and were analyzed with GraphPad Prism software (v. 5.0a; GraphPad Software, La Jolla, CA). Comparisons between various individuals were performed with the Mann-Whitney *U* test or unpaired *t*-test, whereas comparisons between the same individuals were performed with the Wilcoxon matched-pair *t*-test or paired *t*-test when appropriate. Correlations between variables were evaluated with the Spearman rank correlation test. For all the tests, a two-sided *P* value < 0.05 was considered to be significant. Statistical significance is indicated as follows: ^∗∗∗^*P* < 0.001, ^∗∗^*P* < 0.01, and ^∗^*P* < 0.05.

## 3. Results

### 3.1. Changes in Lymphocyte Subsets Differ in Different Types of Pancreatitis

**Figure 1:**
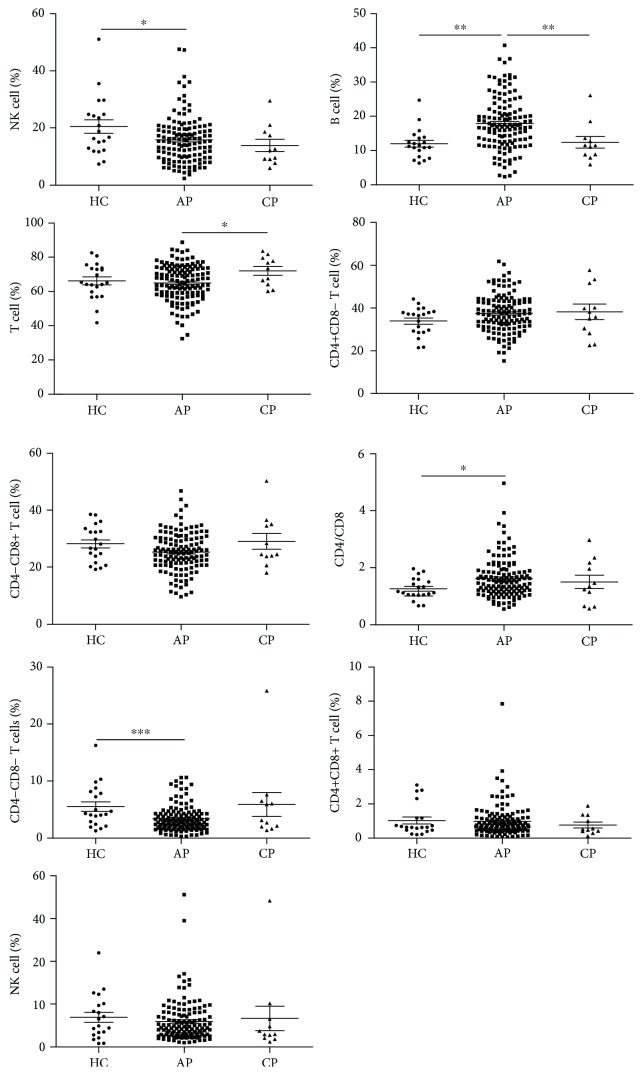
The frequency of peripheral lymphocyte subsets in patients with AP or CP. Pooled data indicating the peripheral lymphocyte subsets (B, NK, T, CD4+ T, CD8+ T, CD4/CD8, DN T, DP T, and NKT) as a proportion of total lymphocytes. ^∗∗∗^*P* < 0.001, ^∗∗^*P* < 0.01, and ^∗^*P* < 0.05.

**Figure 2:**
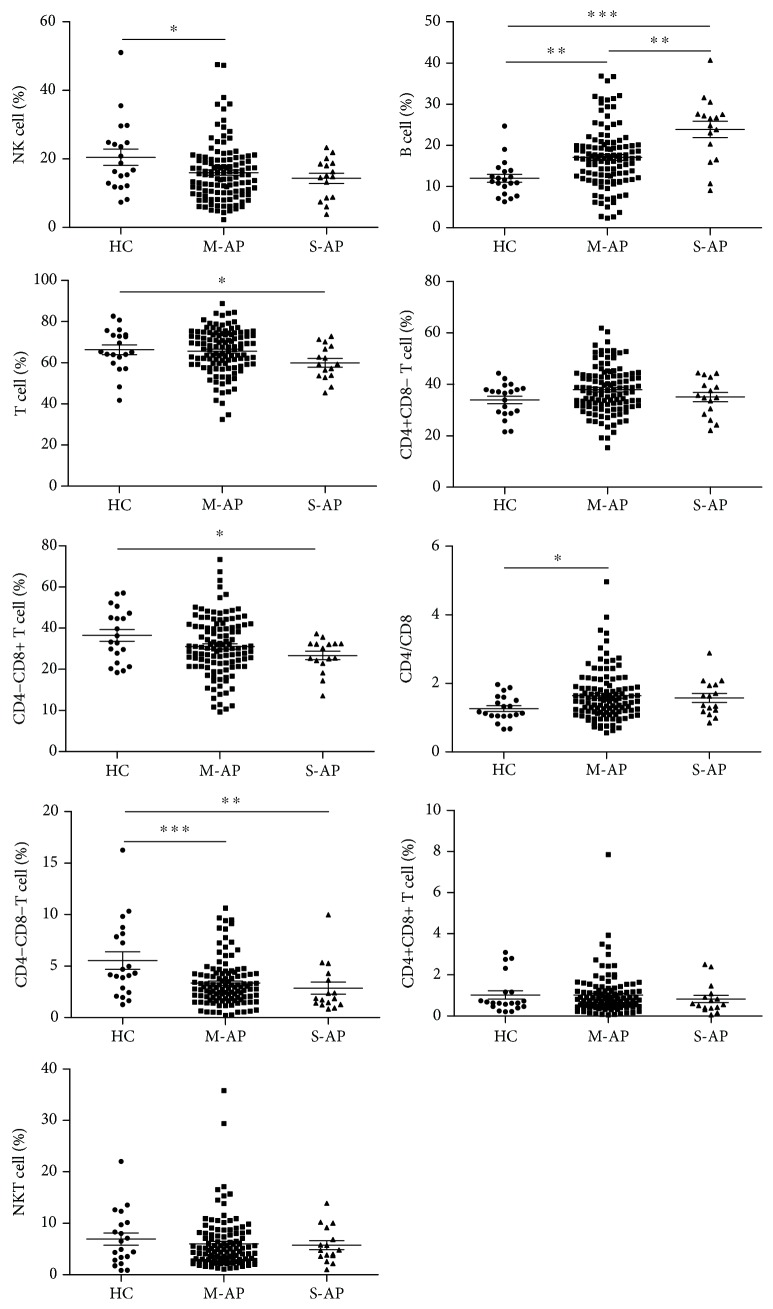
The frequency of peripheral lymphocyte subsets in patients with M-AP or S-AP. Pooled data indicating the peripheral lymphocyte subsets (B, NK, T, CD4+ T, CD8+ T, CD4/CD8, DN T, DP T, and NKT) as a proportion of total lymphocytes.

**Figure 3:**
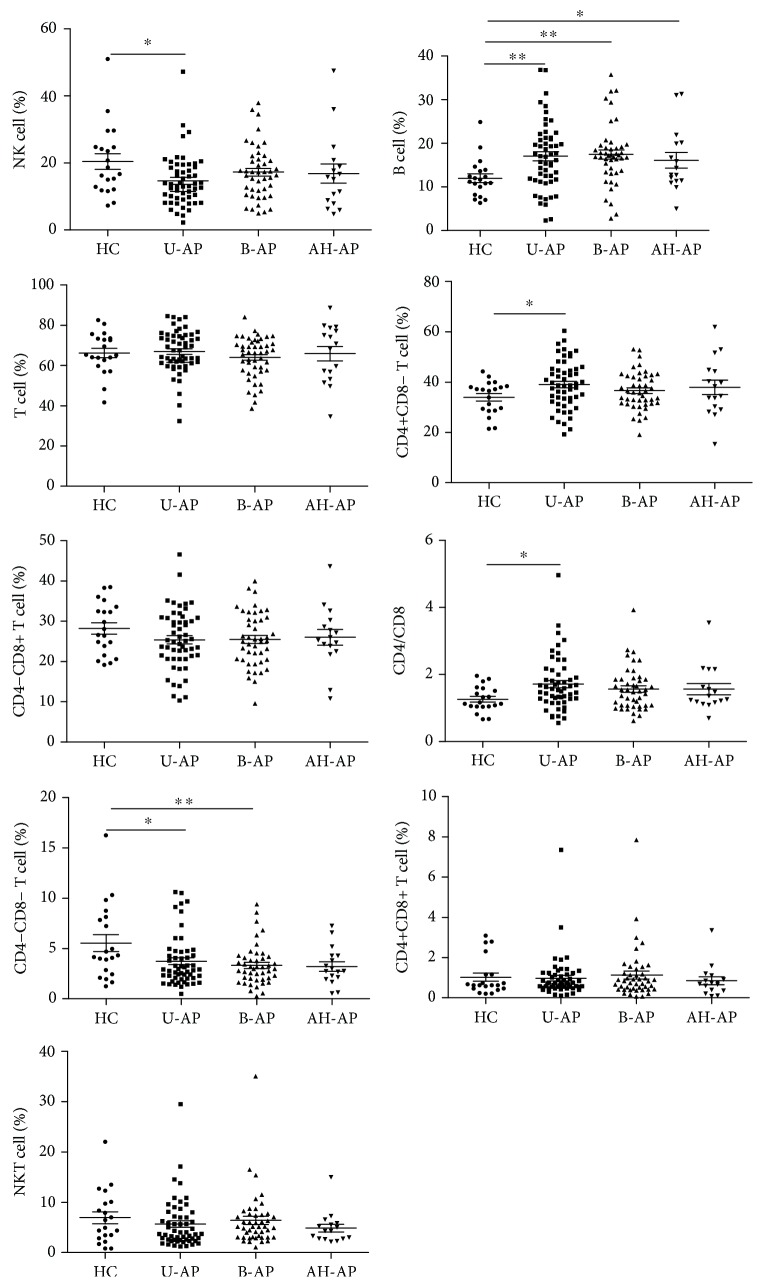
The frequency of peripheral lymphocyte subsets in patients with AP caused by different etiologies. Pooled data indicating the peripheral lymphocyte subsets (B, NK, T, CD4+ T, CD8+ T, CD4/CD8, DN T, DP T, and NKT) as a proportion of total lymphocytes.

Firstly, we detected the frequency and absolute number of peripheral lymphocyte subsets in patients with pancreatitis at the time of admission. We found that in all types of pancreatitis including AP, CP, M-AP, S-AP, biliary-AP (B-AP), unspecified-AP (U-AP), and alcohol and/or hyperlipidemia-AP (AH-AP) with the exception of B cells in S-AP and AH-AP, the absolute number of all other lymphocyte subsets was significantly reduced (Table 4), whereas the frequency change of lymphocytes varies depending on the type of pancreatitis. In patients with AP, B cell frequency and the ratio of CD4/CD8 were significantly elevated while NK cell frequency and DN T cell frequency were significantly reduced. There was no significant change in all lymphocyte subsets in patients with CP compared with HC subjects (Figure 1). Changes in frequency of all lymphocyte subsets in patients with M-AP were the same as those in patients with AP. In patients with S-AP, in addition to an increase in B cell frequency and a decrease in DN T cell frequency, the frequency of total T cells and CD8+ T cells was also significantly reduced when compared with HCs (Figure 2). Analyzing the frequency change of lymphocyte subsets in patients with AP caused by diverse etiologies, we found that only B cell frequency was elevated in patients with AH-AP. In addition to an increased B cell frequency, there was also a decreased DN T cell frequency in B-AP patients. In U-AP patients, in addition to the same changes in lymphocyte subsets as in B-AP and AH-AP patients, there were also a decrease in NK cell frequency and an increase both in CD4+ T cell frequency and the ratio of CD4/CD8 (Figure 3).

### 3.2. The Frequency of B Cells and NK Cells Is Positively Correlated with AP-Related Diagnostic Indicators

**Figure 4:**
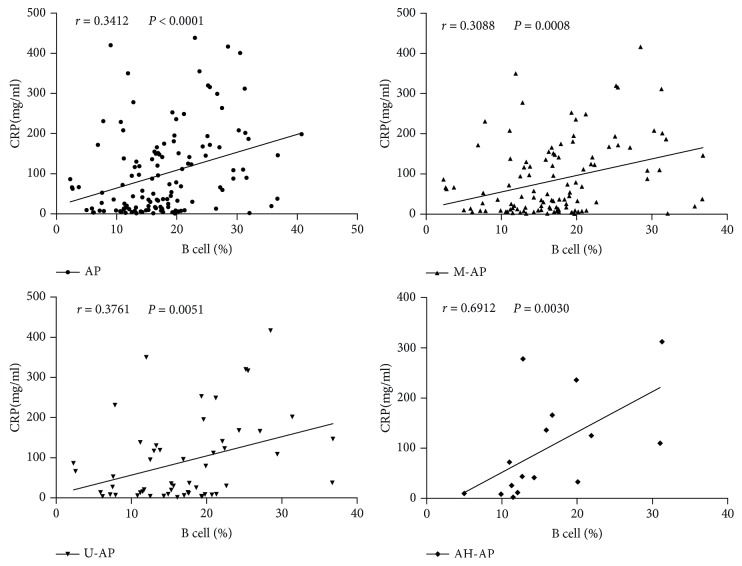
Correlations between B cell frequency and pancreatitis-related diagnostic indicator (CRP) in patients with AP. These data indicate that B cell-frequency and serum CRP concentration have a good positive correlation.

**Figure 5:**
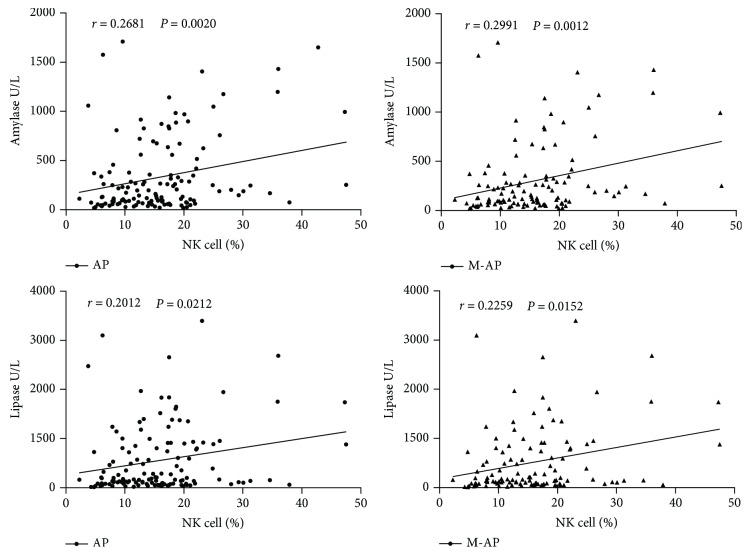
Correlations between NK cell frequency and pancreatitis-related diagnostic indicators (amylase and lipase) in patients with AP. These data indicate that NK cell-frequency and serum amylase and lipase activity concentration have a good positive correlation in patients with M-AP.

To investigate the value of changes in peripheral lymphocyte subsets on patients with AP, we made a correlation analysis between frequency changes in lymphocyte subsets and AP closely related diagnostic indicators (including CRP, amylase, and lipase). We found that except in B-AP and S-AP, B cell frequency and serum CRP concentration showed a good positive correlation in all other types of AP, including M-AP, U-AP, and AH-AP (Figure 4). NK cell frequency correlated well with amylase and lipase activity concentration in AP and M-AP (Figure 5), whereas frequency changes in other lymphocyte subsets did not show significant correlation with these indicators (data not shown). These data suggest that changes in B cells and NK cells may be closely correlated with the pathological lesions of AP and help to diagnose AP more accurately.

### 3.3. Changes in B Cell Frequency and CD4+ T Cell Absolute Number May Be Able to Reflect Therapeutic Effects of Patients with AP in Time

**Figure 6:**
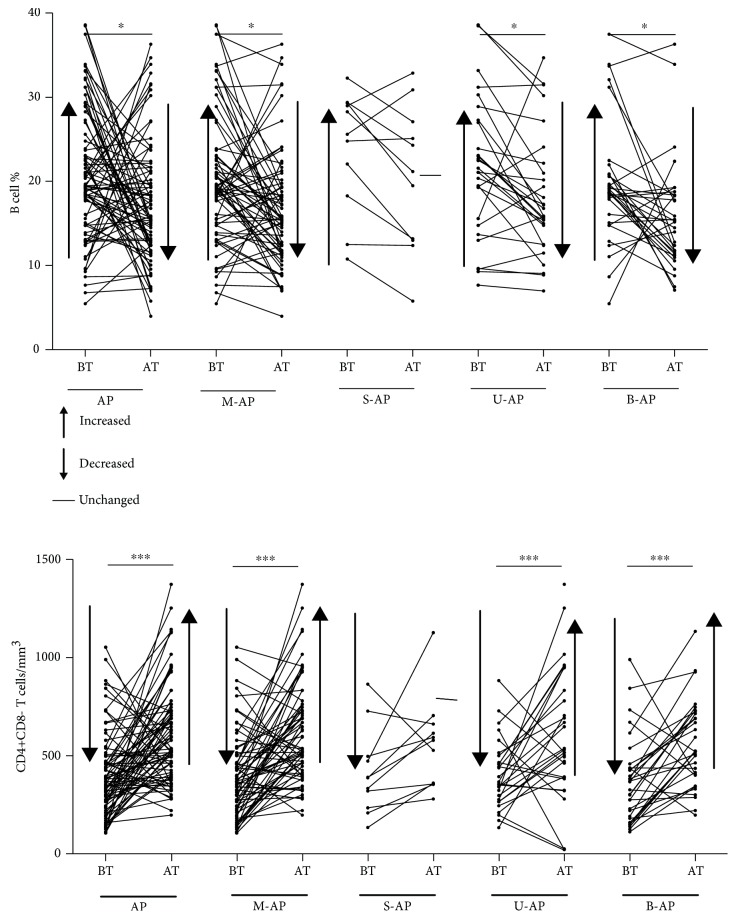
The changes of peripheral lymphocyte subsets (B cells and CD4+ T cells) before and after treatment in patients with AP. The arrow next to BT represents the trend of lymphocyte changes when comparing BT with HC. The arrow next to AT represents the trend of lymphocyte changes when comparing AT with BT.

The activity concentration of serum amylase and lipase may return to be normal after a few days due to their short half-life under untreated conditions; thus, these two indicators cannot accurately reflect the therapeutic effect of patients with AP. Therefore, we analyzed the feedback function of changes in peripheral lymphocyte subsets after short-term treatment on the therapeutic effect of patients with AP. We randomly selected 79 patients from the 131 AP patients enrolled and detected and analyzed their peripheral lymphocyte changes before (BT) and after (AT) treatment. We found that after a 7-day standard treatment, the patients' condition did improve (judging through imaging and other biochemical indicators), but only B cell frequency and CD4+ T cell absolute number in patients with AP (including M-AP, B-AP, and U-AP) tended to be normal (Figure 6). Besides, after 7 days of treatment, T cell frequency in S-AP, DN T cell absolute number in M-AP and CD8+ T cell absolute number in both U-AP and M-AP tended to be normal, while other lymphocyte subsets had no significant change. The change trend of lymphocyte subsets before and after treatment is summarized in Table 5. These data suggest that changes of B cell frequency and CD4+ T cell absolute number may be able to provide more accurate feedback for early therapeutic effects and disease control in patients with AP.

### 3.4. The Frequency of B Cells and NK Cells Is Positively Correlated with Hospitalization Time in Patients with M-AP

**Figure 7:**
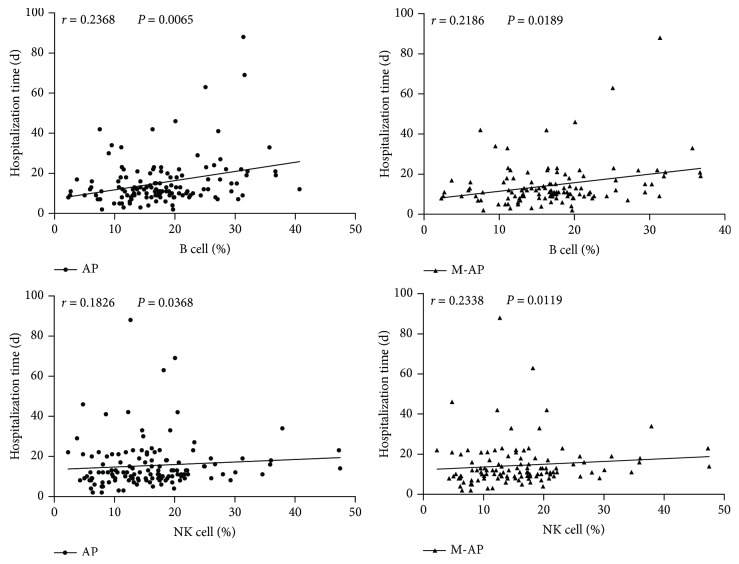
Correlations between the frequency of peripheral lymphocyte subsets (B and NK cells) at admission and hospitalization time in patients with AP. These data suggest that the frequency of B cells and NK cells is positively correlated with hospitalization time in patients with M-AP.

Furthermore, to study the relationship between the change of lymphocyte subsets and the recovery speed of AP patients, we performed a correlation analysis between the frequency of lymphocytes at admission and hospitalization time in AP patients. Through analysis, we found a significant positive correlation between the hospitalization time and the frequency of B cells and NK cells at admission in patients with M-AP (Figure 7), while the frequency of other lymphocyte subsets had no significant correlation with hospitalization time in all types of AP (data not shown). These data suggest that B cells and NK cells may have a more significant effect on pathological state and recovery speed of patients with M-AP.

## 4. Discussion

Deregulation of the immune system is one of the major mechanisms responsible for the death caused by the various types of pancreatitis (including AP and CP). The role of lymphocytes in this phenomenon has been partially investigated [12–17]. However, the impact of changes in peripheral lymphocyte subsets on the diagnostic value, feedback function of disease control, and hospitalization time of patients with AP or CP remains unclear. Therefore, we performed broad and complicated analyses about peripheral lymphocyte subsets and pancreatitis-related diagnostic indicators (amylase, lipase, and CRP) in patients with AP of different severity and etiologies at different time points. Furthermore, we analyzed the correlation between lymphocyte subset changes on admission and hospitalization time in patients with AP. Firstly, we found no change in the absolute number of B cells in patients with S-AP or AH-AP at admission, which was the same as the previous report [7], while other lymphocytes were significantly reduced in all types of AP. As for the frequency change of peripheral lymphocyte subsets, only B cells were significantly increased in various types of AP regardless of the etiologies. The depletion of B cells was slower than other lymphocytes probably due to a less apoptosis [7]. In contrast to B cells, the frequency of DN T cells was decreased in all types of AP except in AH-AP. We do not understand exactly the mechanism that causes this phenomenon in patients with AP. However, it has been found that DN T cells isolated from lpr/lpr mice and from patients with lymphoid proliferative diseases due to germline mutations in Fas or Fas ligand (Fas-L) both abnormally expressed B cell antigen B220 [18]. Li and her colleagues suggested that converted DN T cells could suppress B cell proliferation and induce apoptosis through perforin in a manner of cell-cell contact ex vivo [19]. These findings may help explain why B cell frequency increased when DN T cells decreased in many types of AP. Unexpectedly, the frequency of NK cells decreased in M-AP and U-AP but unchanged in S-AP and other types of AP. Migration to the site of inflammation may result in a decrease of peripheral NK cells, which has recently been described in experimental models of adenovirus-mediated AP [20]. Interestingly, in patients with CP, the absolute number of all lymphocyte subsets decreased but the frequency was unchanged which may be caused by prolonged condition of patients with CP, the body reached a relatively stable immune status, and the proportion of lymphocyte subsets tended to be balance.

CRP is a sensitive inflammatory marker widely used to predict the severity of AP [21, 22]. In this study, we analyzed the correlation between the frequency of peripheral lymphocyte subsets and serum CRP concentration. Through analysis, we found that in all types of AP except in B-AP, B cell frequency was positively correlated with serum CRP concentration. Therefore, peripheral B cell frequency and serum CRP concentrations may be able to synergistically used to assist in diagnosis and determine the severity of patients with AP. Amylase and lipase act as specific diagnostic indicators for AP and CP and have a good correlation with pancreatitis [2, 9]. Therefore, we also analyzed the correlation between the frequency of lymphocyte subsets and serum activity concentration of amylase and lipase in patients with AP at admission. We found that peripheral NK cell frequency was positively correlated with serum activity concentration of amylase and lipase, so it is possible for NK cells to diagnose AP more accurately when together with amylase and lipase.

In the course of treatment, it is necessary to have indicators to reflect the therapeutic effects of pancreatitis in time; the current methods are still mostly limited to imaging and other biochemical indicators due to the short half-life of amylase and lipase. Besides, the feedback function of lymphocytes for early treatment of AP remains unclear [9]. Therefore, we would like to know whether changes in the frequency and absolute number of peripheral lymphocytes could reflect the early treatment effect of AP patients in time. By analysis, we found that after a short-term treatment, the change of B cell frequency and CD4+ T cell absolute number tended to be normal in all types of AP except in S-AP which possibly due to insufficient patients studied in S-AP (11 patients). Therefore, we believe that B cell frequency and CD4+ T cell absolute number may be able to cooperate with other imaging studies and biochemical indicators to reflect therapeutic effect of AP in time.

There are numerous factors that can affect the recovery speed and prognosis of patients with AP. Some studies have shown that obestatin can accelerate the recovery speed of AP caused by ischemia-reperfusion in rats [23], and silencing Mist1 gene expression is crucial for the recovery of AP [24]. We would like to find out if changes in peripheral lymphocytes could affect the recovery speed of patients with AP. Therefore, we analyzed the correlation between the change of peripheral lymphocyte subsets and the hospitalization time in patients with AP. Through analysis, we found that the frequency of NK cells and B cells was closely correlated with the length of hospital stay in patients with M-AP.

In summary, changes in lymphocyte subsets differ in different types of pancreatitis. In almost all types of AP, B cell frequency may be able to combine with serum CRP concentration to determine the severity of AP and to match the absolute number of CD4+ T cells to reflect the treatment effect of AP in time. In patients with M-AP, NK cell frequency may be able to cooperate with amylase and lipase to act as an auxiliary diagnosis indicator to diagnose M-AP more accurately. Furthermore, the frequency of NK cells and B cells was positively correlated with hospitalization time in patients with M-AP. These data suggest that B cells and NK cells closely correlate with the condition of patients with AP.

## Figures and Tables

**Table 1:** Comparison of demographic characteristics and several important laboratory parameters between patients with AP and CP.

	HC (*n* = 20)	AP (*n* = 131)	CP (*n* = 11)	P
Male/female	8/12	84/47	8/3	
Age	47.60 ± 2.552	48.79 ± 1.483	55.82 ± 3.875	*P* = 0.097
Predisposing etiology factors				
Alcohol		5		
Hyperlipidemia		11		
Biliary		45		
Unspecified		70	11	
CRP (mg/mL)	2.705 ± 0.5638	99.75 ± 9.240	49.58 ± 20.02	*P* = 0.089
Amylase (U/L)	58.77 ± 7.253	329.1 ± 32.53	249.1 ± 140.1	*P* = 0.111
Lipase (U/L)	21.88 ± 4.355	552.3 ± 60.70	180.6 ± 109.5	*P* = 0.003
Hospitalization time (d)	0	15.44 ± 1.070	18.82 ± 7.606	*P* = 0.957

P: AP vs. CP; AP: acute pancreatitis; CP: chronic pancreatitis; HC: health controls. Values are presented as mean ± SEM.

**Table 2:** Comparison of demographic characteristics and several important laboratory parameters between patients with M-AP and S-AP.

	M-AP (*n* = 115)	S-AP (*n* = 16)	P
Male/female	73/42	11/5	
Age	49.24 ± 1.564	44.38 ± 4.658	*P* = 0.133
Predisposing etiology factors			
Alcohol	5		
Hyperlipidemia	11		
Biliary	45		
Unspecified	54	16	
CRP (mg/mL)	84.34 ± 8.478	210.5 ± 34.64	*P* < 0.001
Amylase (U/L)	303.8 ± 34.13	510.8 ± 94.63	*P* = 0.087
Lipase (U/L)	517.2 ± 63.40	804.8 ± 192.4	*P* = 0.080
Hospitalization time (d)	14.46 ± 1.070	22.50 ± 3.873	*P* = 0.007

P: M-AP vs. S-AP; M-AP: mild AP; S-AP: severe AP. Values are presented as mean ± SEM.

**Table 3:** Comparison of demographic characteristics and several important laboratory parameters between before and after treatment in patients with AP.

	BT	AT	P
Male/female	55/24		
Age	50.85 ± 1.788		
AP (*n* = 79)			
CRP (mg/mL)	119.0 ± 13.26	56.29 ± 7.575	*P* < 0.0001
Amylase (U/L)	444.7 ± 48.00	108.4 ± 10.68	*P* < 0.0001
Lipase (U/L)	757.1 ± 90.50	155.9 ± 16.77	*P* < 0.0001
Hospitalization time (d)	17.39 ± 1.302		
*Severity of AP*			
M-AP (*n* = 68)			
CRP (mg/mL)	104.4 ± 12.34	42.25 ± 5.865	*P* < 0.0001
Amylase (U/L)	434.0 ± 52.94	112.1 ± 12.10	*P* < 0.0001
Lipase (U/L)	733.1 ± 97.62	154.5 ± 18.03	*P* < 0.0001
Hospitalization time (d)	16.74 ± 1.420		
S-AP (*n* = 11)			
CRP (mg/mL)	231.7 ± 46.82	138.9 ± 31.72	*P* = 0.0682
Amylase (U/L)	592.1 ± 110.5	91.55 ± 17.51	*P* = 0.0137
Lipase (U/L)	1038 ± 243.2	142.8 ± 33.67	*P* = 0.0098
Hospitalization time (d)	22.82 ± 2.885		

P: BT vs. AT; BT: before treatment; AT: after treatment. Values are presented as mean ± SEM.

**Table 4:** Absolute number of lymphocyte subsets when patients on admission.

Cell (/*μ*L)	HC	AP	CP	M-AP	S-AP	B-AP	U-AP	AH-AP
Total T	1733 ± 197.7	791.7 ± 32.35^c^	942.2 ± 111.5^b^	805.8 ± 34.89^c^	668.9 ± 76.36^c^	718.1 ± 55.29^c^	851.5 ± 47.51^c^	898.4 ± 108.9^c^
CD4+ T	875.4 ± 98.43	454.4 ± 19.16^c^	517.1 ± 86.28^a^	461.8 ± 20.56^c^	379.9 ± 50.12^c^	404.9 ± 30.46^c^	492.7 ± 28.91^c^	518.1 ± 66.63^b^
CD8+ T	759.3 ± 101.8	311.1 ± 14.72^c^	365.2 ± 49.26^b^	316.3 ± 16.05^c^	248.8 ± 27.40^c^	290.2 ± 27.11^c^	325.4 ± 21.31^c^	358.9 ± 48.24^b^
DP T	29.08 ± 7.666	11.40 ± 0.9260^c^	8.645 ± 1.863^a^	11.86 ± 0.9784^c^	8.622 ± 2.098^b^	12.84 ± 2.164^b^	11.63 ± 1.554^b^	10.97 ± 2.526^a^
DN T	140.8 ± 25.89	42.25 ± 3.106^c^	74.49 ± 28.45^a^	41.67 ± 3.148^c^	30.62 ± 6.084^c^	38.79 ± 4.834^c^	49.04 ± 5.598^c^	41.31 ± 6.445^c^
B	307.4 ± 36.52	210.6 ± 11.10^b^	157.7 ± 33.58^b^	204.1 ± 11.64^b^	253.1 ± 34.66	184.6 ± 15.93^c^	213.3 ± 16.52^b^	228.1 ± 43.88
NK	659.8 ± 191.5	184.8 ± 11.55^c^	163.1 ± 23.06^c^	190.1 ± 12.85^c^	141.9 ± 19.01^c^	191.9 ± 24.24^c^	182.1 ± 15.57^c^	212.0 ± 35.15^b^
NKT	186.9 ± 41.74	70.12 ± 5.061^c^	79.00 ± 29.00^a^	70.79 ± 5.524^c^	59.82 ± 10.19^b^	71.38 ± 8.638^c^	71.18 ± 8.732^c^	72.51 ± 12.39^a^

^a^ *P* < 0.05 vs. HC; ^b^*P* < 0.01 vs. HC; ^c^*P* < 0.001 vs. HC; B-AP: biliary-AP; U-AP: unspecified-AP; AH-AP: alcohol and/or hyperlipidemia-AP. Values are presented as mean ± SEM.

**Table 5:** A summary of the change trend in lymphocyte frequency and absolute number before and after treatment in AP patients.

	B cells				NK cells				T cells				CD4+ T cells				CD8+ T cells				CD4/CD8		DN T cells				DP T cells				NKT			
	Frequency		Number		Frequency		Number		Frequency		Number		Frequency		Number		Frequency		Number		Ratio		Frequency		Number		Frequency		Number		Frequency		Number	
	BT	AT	BT	AT	BT	AT	BT	AT	BT	AT	BT	AT	BT	AT	BT	AT	BT	AT	BT	AT	BT	AT	BT	AT	BT	AT	BT	AT	BT	AT	BT	AT	BT	AT
AP	↑	↓	↓	—	↓	↓	↓	—	—	—	↓	—	—	—	↓	↑	—	—	↓	↑	↑	↑	↓	—	↓	—	—	—	↓	—	—	—	↓	—
M-AP	↑	↓	↓	—	↓	↓	↓	—	—	—	↓	—	—	—	↓	↑	—	—	↓	↑	↑	↑	↓	—	↓	↑	—	—	↓	—	—	—	↓	—
S-AP	—	—	—	—	—	—	↓	—	↓	↑	↓	—	—	—	↓	—	↓	—	↓	—	—	—	↓	—	↓	—	—	—	↓	—	—	—	↓	—
U-AP	↑	↓	↓	—	↓	↓	↓	—	—	—	↓	—	↑	↑	↓	↑	—	—	↓	↑	—	—	↓	—	↓	—	—	—	↓	—	—	—	↓	—
B-AP	↑	↓	↓	—	—	—	↓	—	—	—	↓	—	—	—	↓	↑	—	—	↓	—	—	—	↓	—	↓	—	—	—	↓	—	—	—	↓	—

BT: BT average compared with HC average; AT: AT average compared with BT average.

## Data Availability

The (raw data) data used to support the findings of this study are available from the corresponding authors upon reasonable request and with permission of all other coauthors.
